# Quantitative Analysis of NF-κB Transactivation Specificity Using a Yeast-Based Functional Assay

**DOI:** 10.1371/journal.pone.0130170

**Published:** 2015-07-06

**Authors:** Vasundhara Sharma, Jennifer J. Jordan, Yari Ciribilli, Michael A. Resnick, Alessandra Bisio, Alberto Inga

**Affiliations:** 1 Laboratory of Transcriptional Networks, Centre for Integrative Biology (CIBIO), University of Trento, Trento, Italy; 2 Chromosome Stability Group; National Institute of Environmental Health Sciences, Research Triangle Park, North Carolina, United States of America; St. Georges University of London, UNITED KINGDOM

## Abstract

The NF-κB transcription factor family plays a central role in innate immunity and inflammation processes and is frequently dysregulated in cancer. We developed an NF-κB functional assay in yeast to investigate the following issues: transactivation specificity of NF-κB proteins acting as homodimers or heterodimers; correlation between transactivation capacity and *in vitro* DNA binding measurements; impact of co-expressed interacting proteins or of small molecule inhibitors on NF-κB-dependent transactivation. Full-length p65 and p50 cDNAs were cloned into centromeric expression vectors under inducible *GAL1* promoter in order to vary their expression levels. Since p50 lacks a transactivation domain (TAD), a chimeric construct containing the TAD derived from p65 was also generated (p50TAD) to address its binding and transactivation potential. The p50TAD and p65 had distinct transactivation specificities towards seventeen different κB response elements (κB-REs) where single nucleotide changes could greatly impact transactivation. For four κB-REs, results in yeast were predictive of transactivation potential measured in the human MCF7 cell lines treated with the NF-κB activator TNFα. Transactivation results in yeast correlated only partially with *in vitro* measured DNA binding affinities, suggesting that features other than strength of interaction with naked DNA affect transactivation, although factors such as chromatin context are kept constant in our isogenic yeast assay. The small molecules BAY11-7082 and ethyl-pyruvate as well as expressed IkBα protein acted as NF-κB inhibitors in yeast, more strongly towards p65. Thus, the yeast-based system can recapitulate NF-κB features found in human cells, thereby providing opportunities to address various NF-κB functions, interactions and chemical modulators.

## Introduction

The nuclear factor-κB (NF-κB) is a ubiquitously expressed family of transcription factors (TFs) that have critical roles in inflammation, immunity, cell proliferation, differentiation and survival [1]. Constitutive activation of these proteins is related to tumor prevalence and various diseases such as arthritis, immunodeficiency and autoimmunity [2]. These proteins are included in the category of rapidly acting, sequence-specific TFs that are present as inactive proteins in the cell and do not require new protein synthesis for activation. The activities of NF-κB proteins are tightly regulated at multiple levels and are influenced by several types of external stimuli as well as internal regulators [3,4]. Among the latter group, the IκB (Inhibitor of NF-κB) family of proteins is prominent among negative regulators of NF-κB activity. IκB associates with NF-κB through noncovalent, stable interactions forming NF-κB/IκB complexes. This interaction masks NF-κB nuclear localization signals, thereby inhibiting NF-κB translocation into the nucleus [5]. External stimuli such as IL-1 (interleukin-1), TNFα (tumor necrosis factor-α) and LPS (bacterial lipopolysaccharide) lead to phosphorylation of IκB by the IκB kinase (IKK) complex protein and subsequently enable nuclear translocation of NF-κB and transcription of the target genes [6,7]. Various pharmacological inhibitors act as direct or indirect inhibitors of NF-κB activity *in vitro* or in mammalian systems. Ethyl pyruvate (EP) directly inhibits NF-κB transactivation by targeting the DNA binding ability of p65 [8]. The small molecule BAY 11–7082 (BAY) has an indirect effect on NF-κB by inhibiting the IκB kinase (IKK) [9,10] or suppressing its activation [11].

The NF-κB family can be divided into two subfamilies: type I (NF-κB1/p50 and NF-κB2/p52) and type II (p65/RELA, RELB and C-Rel). Structurally, the conserved N-terminal region of NF-κB proteins share a sequence homology across all the subunits that is termed Rel Homology Domain (RHD) [12,13] and is responsible for subunit dimerization, sequence-specific DNA binding and nuclear localization. The carboxy-terminal region comprises the transactivation domain (TAD) but is absent in p50 and p52 subunits. These two TAD deficient subunits can activate transcription only when they form heterodimers with a type II subunit or as homodimers in complex with other co-factors. Therefore, NF-κB dimers composed only of p50 and/or p52 subunits fail to activate transcription. The five NF-κB subunits can combine in pairs to produce up to 15 distinct functional NF-κB dimers [14]. Nevertheless, the physiological existence and relevance of all 15 dimers is not completely understood. The p50/p65 heterodimer is the most prevalent and well-studied NF-κB family dimer [14]. The p50 subunit can contribute to p65-mediated transcription, while p50 homodimers may have a repressive effect on NF-κB target gene expression [15]. Some of the NF-κB dimers are rarely observed *in vivo* such as p65/RelB and c-Rel/RelB [16].

NF-κB homo- or hetero-dimers target a loose consensus sequence of 9–11 base pairs embedded in promoter or enhancer regions of target genes, referred to as κB binding site or κB Response Element (κB-RE). The general motif of this consensus sequence is 5’-GGGRNWYYCC-3’ (R = purine, N = any nucleotide, W = adenine or thymine, and Y = pyrimidine) [13]. Each NF-κB monomer occupies half of the κB-RE. NF-κB homo or heterodimers exhibit distinct DNA binding preferences towards specific κB-REs. The optimal DNA binding motifs for p50 and p65 homodimers based on *in vitro* selection are GGGGATYCCC and GGGRNTTTCC, respectively [17]. Distinct physical contacts along the 10-base-pair κB RE by NF-κB p50 homodimer or p65 homodimer have been identified through crystal structure analyses [18],[19]. The exact nature and mechanism of interactions between NF-κB and κB-RE sequences responsible for changes in transactivation specificities are not clearly understood. A single nucleotide change within κB-RE sequences can dramatically alter binding affinity, thereby impacting NF-κB-dependent target gene expression [20,21]. In a previous study, the transactivation potential of a κB-RE was revealed to be strongly influenced by the central base pair, which also impacts recruitment of p52 and p65 homodimers [22]. It has been proposed that NF-κB family transactivation specificity is not uniquely coded in the κB-RE sequence, suggesting participation of other cofactors in subunit specificity at NF-κB target gene promoters [23].

To investigate the role of κB-RE sequences in mediating transactivation specificity in the absence of other endogenous regulatory regions that might influence κB-RE and NF-κB dynamics, we developed a versatile yeast-based NF-κB functional assay inspired by our previous work on the p53 family of transcription factors [24–26]. Since p50/p65 heterodimer is the most prevalent and well-studied dimer member of NF-κB family, we focused our attention on these proteins. Using several κB-REs selected from endogenous target sites, or differing by single nucleotide substitutions, we have employed *in vivo* quantitative analysis to address a) transactivation specificity of NF-κB proteins acting as homodimers or heterodimers; b) correlation between transactivation capacity and DNA binding measurements *in vitro*; c) impact of protein interactors, namely IκBα; and d) impact of small-molecule inhibitors on transactivation by NF-κB proteins.

## Materials and Methods

### Construction of yeast reporter strains containing κB-REs regulating the expression of the Firefly cDNA

A panel of isogenic yeast reporter strain containing different version of single copies of the decameric κB-RE sequences, or two copies in tandem was constructed using single strand targeting oligonucleotides (Eurofins MWG Operon, Ebersberg, Germany), at the chromosomal XV locus containing a minimal *CYC1* promoter driving the expression of the *Firefly* luciferase cDNA. These experiments were developed following a previously published approach [24,25] that is an adaptation of the “*delitto perfetto*” approach to *in-vivo* site-directed mutagenesis [27,28] and starts with the yLFM-ICORE strain [25,29–31]. The protocol utilizes single-strand oligonucleotides that contain a desired κB-RE and exploits a triple-marker cassette positioned in the yLFM-ICORE strain near the *CYC1* promoter. The cassette contains a counter-selectable gene (*URA3*), a reporter gene (*kanMX4*), and the I-SceI homing endonuclease along with its unique 18nt recognition site. The latter gene allowed us to engineer a unique double-strand break at the site where the cassette is cloned, which in turn leads to high efficiency oligonucleotide targeting *via* homologous recombination mediated by short (30nt) homology tails in the oligonucleotide sequence. These tails correspond to the sequence flanking the I-SceI target site placed on the chromosome, while the desired κB-RE sequence is at the center of the oligonucleotide sequence. Oligonucleotide targeting events were selected exploiting the counter-selectable and reporter markers of the ICORE cassette and correct reporter strains’ construction was confirmed by colony PCR across the engineered chromosomal region, followed by DNA direct sequencing (BMR Genomics, Padua, Italy).

### Construction of p65 and p50 inducible yeast expression vectors

Inducible expression in yeast of NF-κB family proteins was achieved with a *GAL1-10* promoter using either a pTSG- (TRP1) or a pLSG- based (LEU2) vector [24,30]. Cloned sources of NF-κB protein cDNAs were a generous gift from Dr. Michael Karin (University of California at San Diego). Plasmid construction was achieved by standard restriction/ligation approaches and clones obtained upon transformation of competent *E*. *coli* cells were checked by DNA sequencing (BMR Genomics, Padua, Italy). In the case of p50 and RelB, chimeric constructs were also constructed by fusing the region from p65/RelA corresponding to amino acids 302–549 and containing the transactivation domain, to the C-terminal end of either coding sequences, removing the stop codon. These chimeras were obtained by a PCR-based approach using specific primers amplifying the chosen portion of the p65/RelA cDNA and containing homology tails for a gap-repair approach in yeast [32]. The PCR amplicon was then co-transformed together with the pTSG-p65 or pTSG-RelB plasmids linearized using the SalI or NotI enzymes that are present downstream to the cloned cDNA stop codon. Transformants of this gap-repair assay were cultured to extract genomic and plasmid DNA and then used to transform *E*. *coli* competent cells to prepare plasmid DNAs that were then checked by restriction digestion and confirmed by DNA sequencing (BMR Genomics). The resulting plasmids were named pTSG-p50TAD and pTSG-RelBTAD. The latter was however inactive in the yeast-based transactivation assays (data not shown). Plasmid pLSG-p50TAD was constructed starting from pTSG-p50TAD (originally derived from pRS314) and pRS315 [33] swapping the portion of the vector containing the selection marker by PvuI restriction and ligation.

### IκBα cDNA cloning into an *ADH1*-based yeast expression vector

Using a designed set of primers containing flanking 5’ and 3’ homology tails for the *ADH1* promoter region and a *cyc1*-derived terminator sequence, we amplified the human IκBα coding sequence (953 bp) from cDNA obtained from total RNA extraction of human MCF-7 cells to obtain a PCR amplicon that could be used in a gap repair experiment. As receiving vector for this recombination-based cloning we used the centromeric pRS315-derived pLS-Ad vector [24], containing the *LEU2* selectable marker, the constitutive *ADH1* (alcohol dehydrogenase 1 gene) promoter for expressing cDNA of interest and a terminator sequence from the *CYC1* gene to facilitate transcriptional termination. The pLS-Ad was digested with XhoI and SalI and co-transformed in yeast together with the PCR product using the lithium acetate yeast transformation protocol [34]. Transformants were selected on plates lacking leucine (referred to as SDlA). Randomly selected colonies from SDlA plates were used to recover the expected recombinant plasmid through yeast total DNA extraction protocol, transformation into DH5alpha *E*. *coli* competent cells, subsequent DNA extraction (Qiagen, Milan, Italy), followed by digestion with restriction enzymes to select positive clones, which were further verified by DNA sequencing (BMR Genomics, Padua, Italy). Co-transformants (LiAc protocol) of the correct IκB vector clone (LEU2 marked) with an NF-κB expression vector (TRP1 marked) into yLFM-M2 reporter strain were selected on double drop-out plates lacking leucine and tryptophan and containing a high amount of adenine The empty vector pRS315 was co-transformed along with each of the NF-κB expression vectors or with pRS314 to generate an appropriate control.

### Yeast-based Luciferase assays

The newly constructed yLFM-κB-RE strains were transformed with the NF-κB expression vectors. Transformants were then processed for the miniaturized protocol of the luciferase assay we recently developed [24]. Briefly, transformant colonies kept on selective glucose plates were grown for 16 hours, unless otherwise stated, in synthetic liquid media containing raffinose (2%) with or without different concentrations of galactose, which serves as inducer of the *GAL1* promoter driving the expression of the NF-κB proteins. Luciferase activity was measured using the Bright Glo Luciferase assay kit (Promega, Milan, Italy) and expressed either as relative light units (RLU) normalized to optical density (600 nm), subtracting the luminescence obtained by the cells transformed with the empty vector in each reporter strain, or as fold-induction over the empty vector. Experiments included four biological repeats and the results were plotted as mean values and the standard errors of the mean.

### Gene reporter assays in MCF7 cells

Four κB-REs were tested in the human MCF7 cell line. Two copies of the decameric motif were cloned in direct orientation upstream of Firefly luciferase gene within the pGL4.26 vector (Promega), using a pair of complementary oligonucleotides ligated into KpnI/XhoI double-digested plasmid. A unique NdeI restriction site was included 5’ to the κB-REs to facilitate the identification of ligation products. Plasmids were purified and sequenced across the modified region. For gene reporter assays, MCF7 cells were transiently transfected at ~80% confluence using FugeneHD (Promega) with the newly constructed pGL4.26 reporters and the pRL-SV40 control luciferase vector. Twenty-four hours after transfection, cells were treated with the immune-cytokine TNFα (either 10ng/ml or 50ng/ml). When needed, the IKK inhibitor BAY11-7082 was added at the final concentration of 20μM for a total of 8 hours. Dual luciferase assays were performed 4 hours or 8 hours after TNFα treatment, following the manufacturer’s protocol.

### Western Blot assays in yeast and human cells

Yeast transformants were grown for 16 hours in selective medium containing the indicated amounts of galactose to induce the expression of NF-κB cDNAs from the *GAL1*-based vectors. An equivalent amount of cells, based on the culture absorbance measurement (corresponding to 2.5 OD measured at OD_600nm_), was collected by centrifugation. Cells were processed following extraction protocols described previously [35,36] and 15 μl of extracts were loaded on 12% poly-acrylamide gels. Transfer onto nitrocellulose membranes was achieved using the i-Blot semi-dry system (InVitrogen, Life Technologies, Milan, Italy). Specific antibodies directed against p65/RelA TAD domain (clone C-20 Santa Cruz Biotechnology, Milan, Italy) or p53 (clone DO-1, Santa Cruz Biotechnology) were diluted in 1% non-fat skim milk dissolved in PBS-T. PGK1 (Phospho Glycerate Kinase 1) was used as a loading control and immune-detected with a monoclonal antibody (clone 22C5D8, Life Technologies, Milan, Italy). Nuclear and cytoplasmic protein lysates from human MCF7 cells were prepared using NE-PER Nuclear and Cytoplasmic Extraction Kit (Thermo Fisher, Monza, MB, Italy) and quantified using the BCA assay (Thermo Fisher). 20 μg of proteins were loaded onto 12% poly-acrylamide gels. p65 (clone C-20), p50 (clone H119, Santa Cruz), GAPDH (cytoplasmic markers; clone 6C5, Santa Cruz) and Histone 3 (nuclear marker, Ab1791, Abcam) were probed. Immuno-reactive bands were detected using the ECL Select reagent (Amersham, GE Health Care, Milan, Italy) and the ChemiDoc XRS+ documentation system through the ImageLab software (BioRad, Milan, Italy).

## Results

### Development of p65 and p50 expression vectors and reporter yeast strains

We cloned full-length human p65 and p50 cDNAs under the control of the *GAL1* promoter in a centromeric yeast expression vector. This promoter was chosen as it enables a wide variation in protein expression in cells cultured in medium containing raffinose (the carbon source) supplemented with various amounts of galactose to induce *GAL1* transcription [37]. We were confident that p65 could act as a sequence-specific TF in yeast based on a previous report [38] and on the fact that the protein contains a transactivation domain of the acidic class that is reported to be proficient in contacting the yeast transcriptional machinery [39]. However, given that p50 does not contain a transactivation domain, we also generated a chimeric p50-referred to as p50TAD- where the transactivation domain of p65 was fused to the carboxy-terminus of the p50 coding sequence. This provided a unique opportunity for an *in vivo* functional assessment of p50 DNA binding specificity.

To develop isogenic κB reporter strains, we took advantage of the *delitto perfetto* based protocol [24,28] for *in vivo* targeting κB-REs to a chromosomal locus containing a minimal promoter placed upstream of the Firefly luciferase gene [24,28]. The protocol is described in the Material and Methods. Seventeen κB-REs were chosen to sample a wide range of DNA binding affinities that had previously been measured by *in vitro* gel shift assays (see sequences in Table A in S1 File) [21]. The seventeen yeast reporter strains were transformed with p65 or p50 expression vectors and luciferase assays were developed following a miniaturized protocol we previously established for the p53 family of transcription factors [24].

### p65 and p50TAD exhibited differences in relative transactivation capacity and specificity

**Fig 1:**
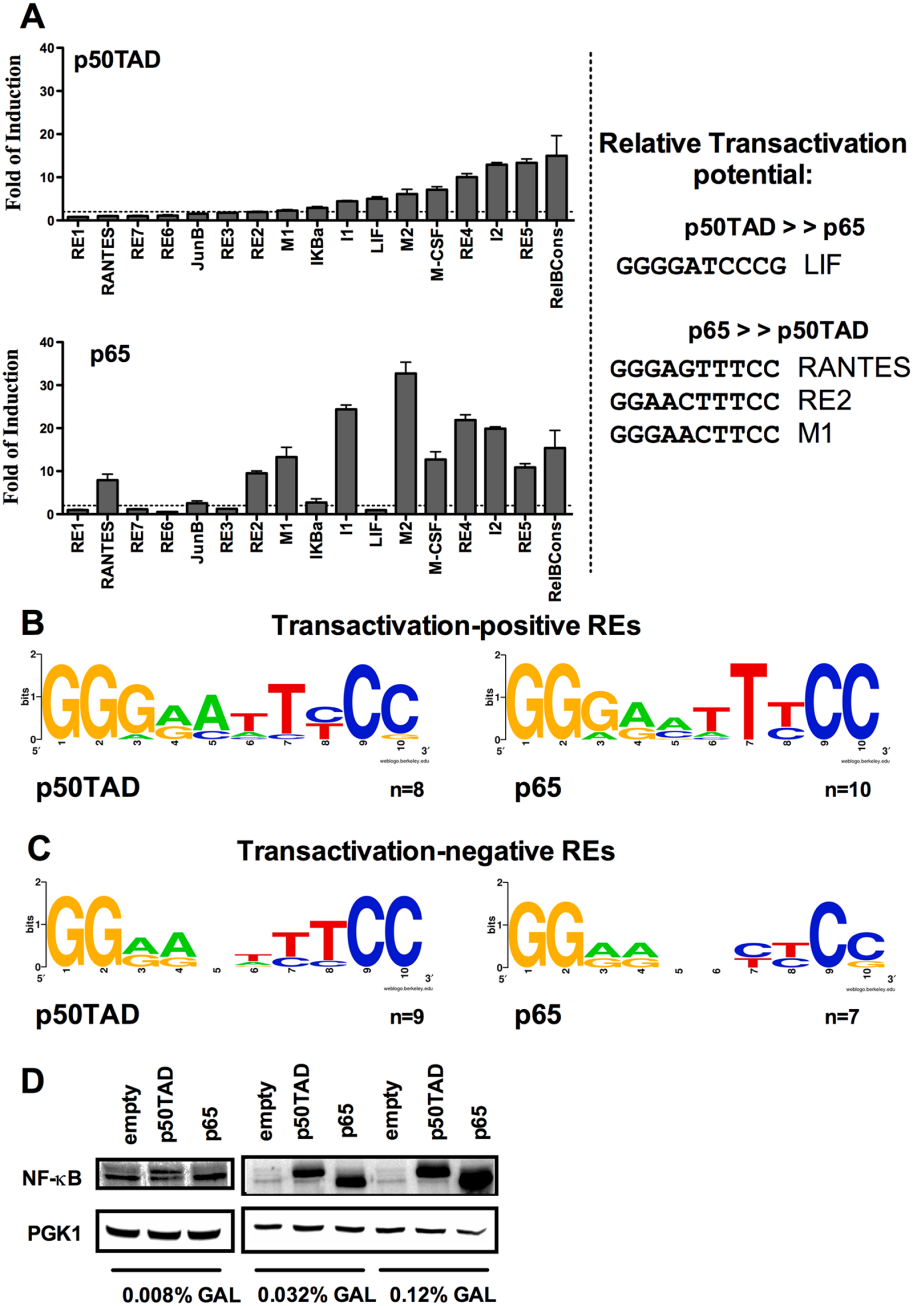
Relative transactivation potential of p50 and p65 homodimers towards a panel of κB Response Elements. **A)** κB target site preferences of p50TAD and p65 homodimers. Luciferase assays were performed to quantify relative transactivation capacity of p50TAD and p65 homodimers towards 17 different κB-REs. Reporter strains were grown in selective media containing 0.032% galactose for 16 hours reaching near stationary phase. For each isogenic reporter strain, the luciferase activity is calculated as fold-induction with respect to the values obtained with empty vector transformants. The average normalized activity and the standard error of four biological repeats are presented. κB-REs are ranked based on increasing transactivation potential with p50TAD. The same rank is used to plot the results obtained with p65 (lower panel). To the right are presented κB-RE sequences that are selectively responsive to either p50TAD or p65 (see text for sequence match to optimized consensus for p50 or p65). **B, C)** Web logo representations of the groups of κB-REs that were active or inactive with p50TAD and p65, respectively. **D)** Western blots presenting the relative expression of p50TAD and p65 proteins at different amounts of galactose. Yeast cells transformed with the *GAL1-*based expression vectors for NF-κB proteins were cultured for 16 hours at the indicated concentrations of galactose. An antibody directed against the p65 transactivation domain, which is also present in the p50TAD construct, was used for immunodetection. PGK1 endogenous protein provides a loading control.

Presented in Fig 1A are the results of transactivation at the seventeen REs following moderate levels of induction of p65 or p50TAD (0.032% gal, as described in Fig 1D). p50TAD activated transcription of eight of the seventeen REs (Fig 1A). This result establishes that p50 can establish specific interactions with target κB-RE sequences when expressed in yeast. p65 was active towards ten κB-REs, but the relative transactivation potentials and pattern of specificities differed remarkably from that of p50TAD (Fig 1A). Five κB-REs were inactive both with p65 and p50TAD. One RE (LIF) was specifically responsive to p50TAD, while three (RANTES, RE2, M1) were selectively responsive to p65. Some of the κB-REs are named from the corresponding human NF-κB target genes. However, in our assay only the decameric κB motif is studied.

Consensus sequences generated using the web logo tool with the transactivation-proficient and deficient κB—REs revealed that mismatches at positions 6 but, surprisingly, also changes at the supposedly permissive position 5 of the decameric G_1_G_2_G_3_R_4_N_5_W_6_Y_7_Y_8_C_9_C_10_ κB—RE impaired transactivation by both proteins (Fig 1B and 1C). Moreover, p50TAD showed a preference for an A in position 5, while a T at position 8 also negatively impacted transactivation. Taken collectively, the results were in agreement with reported differences in p50 and p65 optimal binding sites determined by SELEX [40]. Overall, p65 was a stronger transcription factor compared to p50TAD (both were expressed at similar levels; Fig 1D).

### Both p50TAD and p65 exhibited weak transactivation cooperativity from adjacent κB-REs

**Fig 2:**
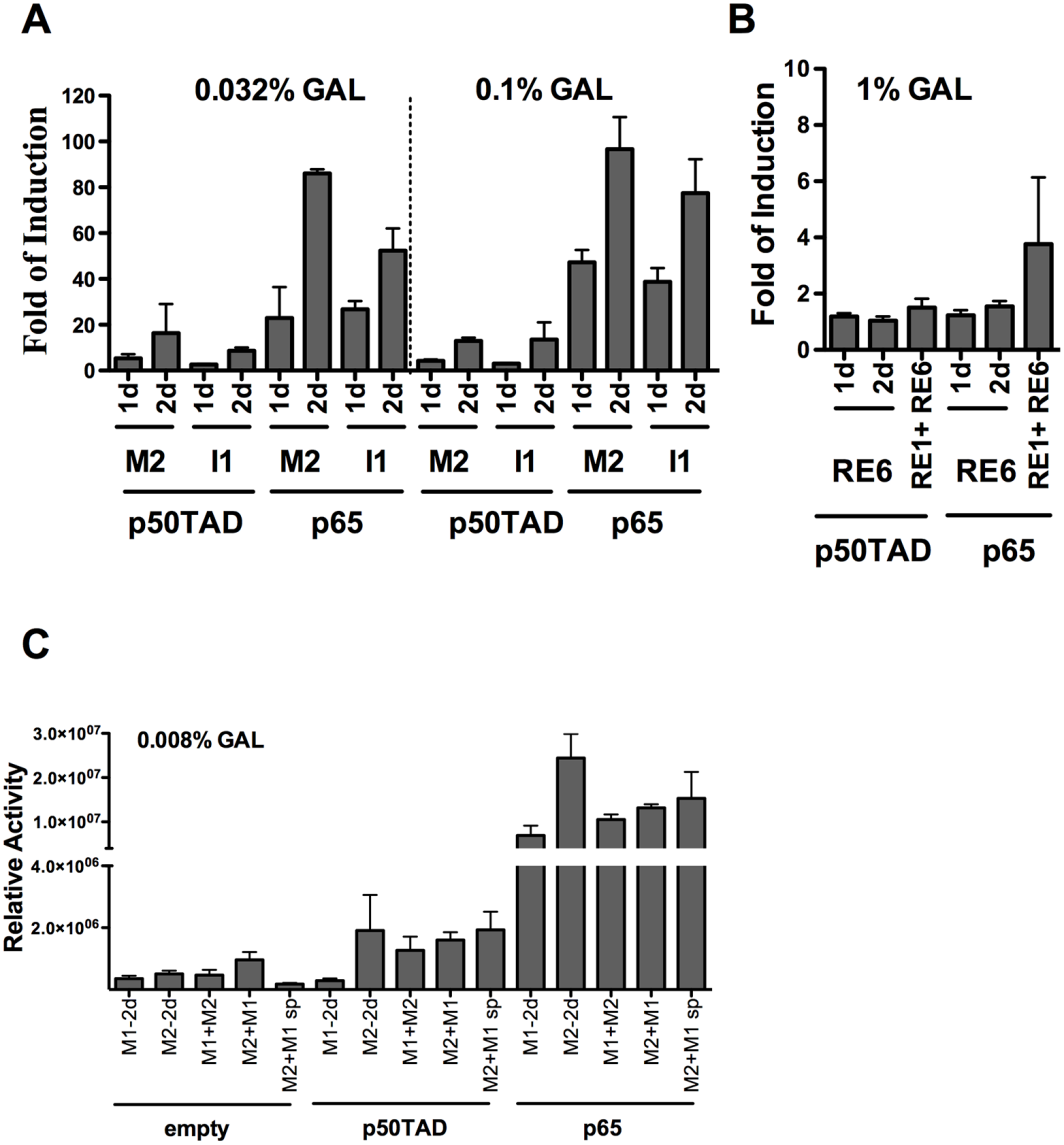
Additive or weak cooperative transactivation between adjacent κB REs. **A)** Yeast-based luciferase assays were performed at moderate to high expression levels of p50TAD or p65. Results were normalized and plotted as in Fig 1. The impact of tandem duplication (2d) of the decameric (1d) κB-RE or the indicated combination of two different κB-REs was evaluated. The REs were chosen based on the results from Fig 1 to include sequences exhibiting different levels of transactivation potentials. **B)** RE1 and RE6 were inactive as decameric κB-REs and there was no transactivation for two decamers in tandem or high expression of NF-κB proteins (up to 1% galactose). **C)** Functional interactions between two different κB-REs derived from the MCP-1 promoter. The M1 and M2 decameric κB-REs exhibited different responsiveness to p50TAD and p65, when examined separately, but are both derived from the MCP-1 promoter and they are located in close distance (19 nucleotide spacer) in the natural context. The combined responsiveness of the M1 and M2 κB-REs was examined, taking into account the impact of the distance between the two decamers and orientation relative to the transcriptional start site. Tandem repeats of M1 or M2 were included as controls. Yeast reporter strains, transformed with the indicated expression vector were cultured for 16 hrs with the indicated low amount of galactose. Relative activity refers to the average light units normalized for cell number (measured by optical density at 600nm). Average and standard error of four biological repeats are presented. The NF-κB-independent reporter activity (empty vector) is also presented as reference. Interestingly, the M2+M1 strains exhibited higher basal level of reporter expression. The M1+M2 sp strain contains the M1 and M2 κB decamers separated by 18 nt as in the human gene (see Table A in S1 File).

Having established that both p65 and the chimeric p50TAD could act as sequence specific TFs in yeast, we explored whether two adjacent κB-REs could lead to synergistic induction of transactivation. Some natural NF-κB target sites contain pairs of κB-REs, whose sequence features can impact on gene transcription [20]. Functional interactions between nearby κB-RE sites have been reported [22] and the potential for cooperative interactions between NF-κB proteins at clustered binding sites has been inferred [41], but not directly addressed under a defined experimental system. The experiments were performed using two different amounts of galactose to titrate the transactivation response. The M2 and I1 REs, which were highly responsive to p65 and moderately responsive to p50TAD, were chosen. Reporter strains containing two adjacent RE copies had an additive effect when analyzed with p50TAD for both κB-REs. A higher amount of galactose (0.1%) did not lead to higher transactivation levels with p50TAD, suggesting that the maximal level of responsiveness for these strains was reached at 0.032% galactose. Instead, p65 showed a galactose-dependent transactivation ability and mainly additive effects for one vs two REs number, with the exception of the M2 RE at the lower galactose concentration (Fig 2A). We also tested RE6 and the combination of RE6 and RE1, which were inactive both with p65 and p50TAD when studied separately. Two copies of the non-responsive RE6 did not lead to any transactivation either with p50TAD or p65 even using very high expression levels (1% galactose) (Fig 2B). The same was true for the combination of RE6 and RE1 except for weak responsiveness to p65.

Next, we investigated *cis*-based interactions among different κB-REs using those from the MCP1 promoter as an example. The responsiveness to NF-κB of this promoter in human cells is mediated by two closely-spaced REs (M1 and M2) that are separated by 19 nt and are located ~ -2.8kb from the transcriptional start site (TSS) [20,22]. As noted above, the M2 κB-RE was highly responsive to p65, while M1 exhibited lower responsiveness. On the contrary, the M1 κB-RE was not responsive to p50, while M2 was weakly responsive. We developed reporter strains containing combinations of M1 and M2 κB-REs, and also examined the impact of the 19nt spacer sequence between them as well as the effect of the relative positioning of the two κB-REs with respect to the promoter of the reporter and the TSS. Strains containing two copies of the M1 or M2 κB-REs were used as controls (Fig 2C).

The non-responsiveness of the M1 RE to p50TAD was confirmed, even when two adjacent copies of this κB-RE are placed upstream the reporter cassette. The reporter strain containing both M1 and M2 showed responsiveness to p50TAD, similar to what was observed with the M2 strain, while the transactivation by p65 was intermediate between the M1 and M2 strains. The M1 κB-RE had a stronger negative impact towards p65 than p50TAD. The relative positioning of the M1 and M2 κB-REs relative to the TSS of the reporter gene did not affect the transactivation potential. Interestingly, the natural 19nt spacer between the M1 and M2 decamers did not impact the transactivation potential. Overall it appears that there is limited functional interaction between adjacent or closely spaced κB-REs for p50- or p65-dependent transactivation in yeast.

### Co-expression of p50 and p65 leads to specific changes in relative transactivation

**Fig 3:**
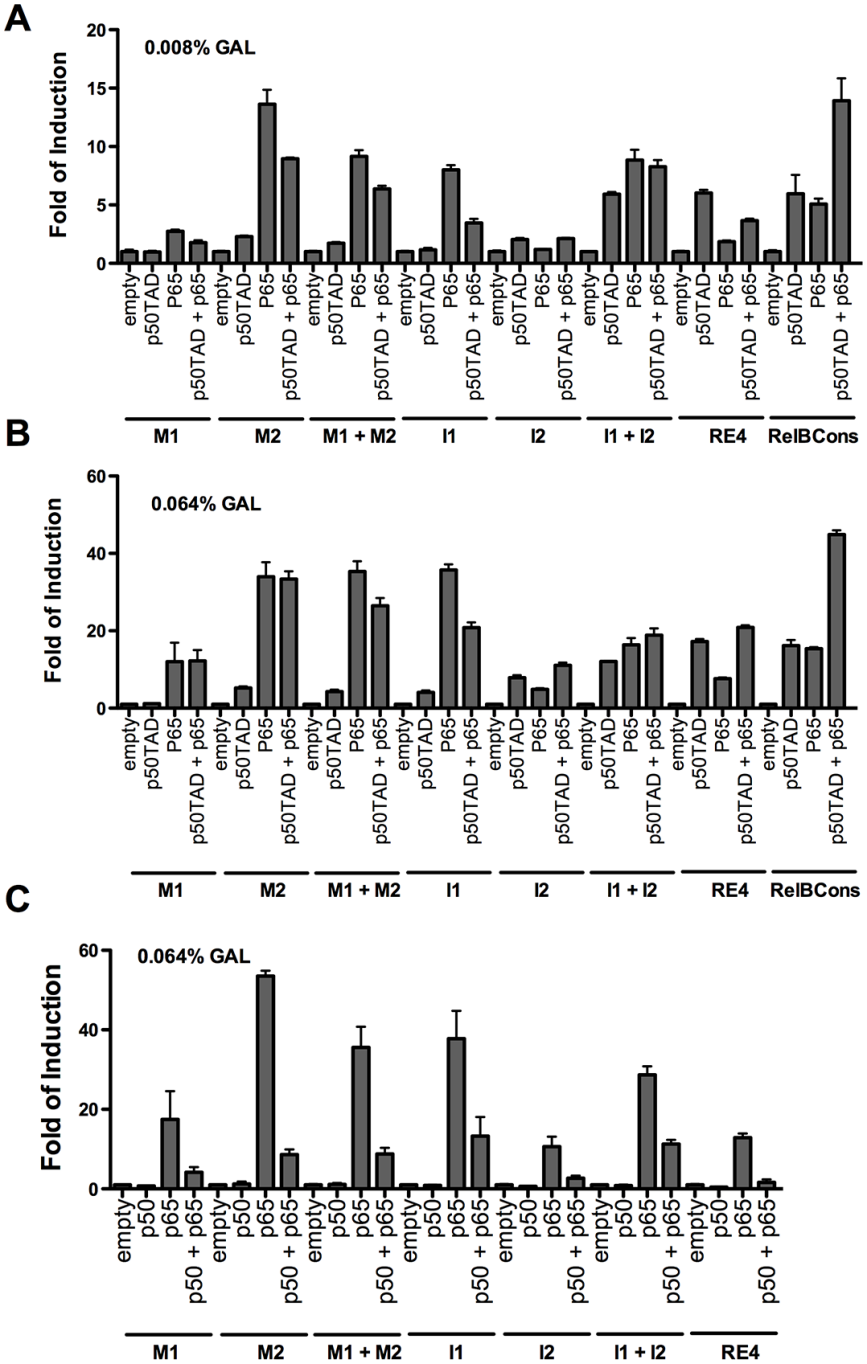
Functional interactions between p50 and p65 towards a panel of κB-REs. **A)** and **B)** Eight different κB-REs, each comprising two adjacent copies of the decameric κB sequences were examined for the transactivation potentials of p50TAD, p65 as well as upon co-expression of both proteins. Cells were grown in lower (0.008%, panel A) and high (0.064%, panel B) levels of galactose. **C)** A non-chimeric p50 construct lacking the TAD domain was also studied at the higher galactose level for all REs, except RelBCons. In all panels, luciferase assays were conducted and results plotted as described in Fig 1.

Since p50 and p65 are normally present at the same time in mammalian cells and the p50/p65 heterodimer is considered a prominent functional complex *in vivo*, we examined p50 and p65 co-expression and transactivation towards the eight κB-REs described in Fig 3A and 3B. Both genes were expressed under the same *GAL1* promoter on different single copy centromere plasmids. Experiments were performed at two levels of NF-κB protein expression where co-expression of p50TAD and p65 resulted in transactivation levels that were approximately an average of those seen with the expression of either protein, with the exception of the RelBCons κB-RE. The relative proportion of homodimers and heterodimers was not determined. In fact, p50TAD or p65 alone led to similar levels of RelBCons κB-RE transactivation but the reporter was much more responsive in the co-expression experiment. The strain with the I1 and I2 κB-REs exhibited similar responsiveness to both p50TAD and p65, but co-expression of these two proteins did not have an impact on the level of transactivation of this promoter. At higher levels of galactose (Fig 3B), the differences between expression of p65 alone or co-expression were lower, with the RelBCons strain maintaining high responsiveness when the two TFs were co-expressed. The experiment was performed also at different time points of NF-κB proteins induction with comparable results (Fig A in S1 File).

We also examined the impact of full-length p50 without the chimeric TAD, expressed alone or combined with p65 protein (Fig 3C). As expected, given the lack of a TAD, p50 was inactive as a transcription factor. However, the presence of the full-length p50 actually led to inhibition of p65-dependent transactivation in the co-expression experiments, much more than could be simply accounted for by the anticipated amount of p50 homodimer. This inhibition could be due possibly to competition for the RE site by p50 homodimers, that can be proposed for the case of I2 or RE4 given the transactivation levels with p50TAD homodimers in those strains (see Fig 3B). For other REs, such as M1 and M2 that are weakly if at all responsive to p50TAD homodimers, it could be inferred that in the co-expression experiments, heterodimers would be preferentially formed and that the p65-p50 dimer would be a weaker transcription factor compared to p65-p50TAD.

### The relative transactivation potentials of selected κB-REs is confirmed in MCF7 cells

**Fig 4:**
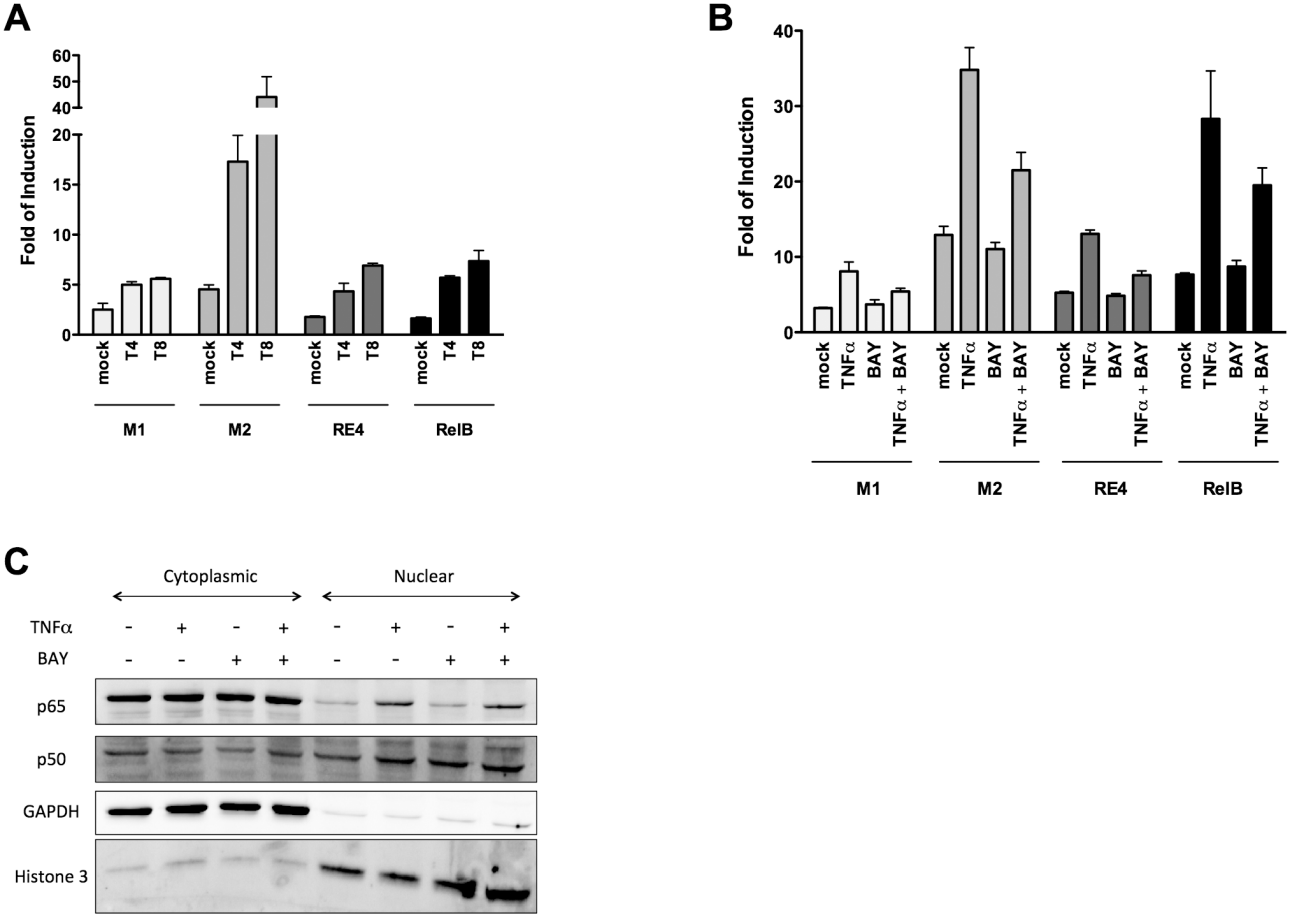
Functional evaluation of four selected κB-REs in MCF7 cells. **A-B)** MCF7 cells were transiently transfected with four pGL4.26 derived vectors containing different κB-REs along with a control pGL4.26 empty vector. Twenty-four hours after transfection cells were treated for 4 or 8 hours with TNFα (10ng/ml for panel A or 50ng/ml for panel B) alone or in combination with BAY11-7082 (20μM for 8 hours, only for panel B). Presented are the average fold-induction relative to the empty pGL4.26 vector and the standard deviation of at least three independent biological replicates. **C)** Western blot analysis showing p50 and p65 protein levels from nuclear-cytoplasmic fractions after the indicated treatments at the following doses: TNFα (50ng/ml) and BAY11-7082 (20μM). GAPDH and Histone 3 were used as reference controls for the cytoplasmic and nuclear fraction respectively.

To examine whether the differences in transactivation capacity observed in yeast were predictive of variable responsiveness in mammalian cells upon NF-κB activation, κB-dependent gene reporter assays were carried out in the human MCF7 cells. We selected 4 different κB-REs whose transactivation potential driven by co-expressed p65 and p50TAD ranked from high (M2, RelBCons) to medium (RE4) and to low (M1) in yeast. Those κB-REs were placed upstream of the Firefly luciferase in the pGL4.26 vector for transient transfection experiments. Twenty-four hours post-transfection cells were treated with 10ng/ml (Fig 4A) or 50ng/ml (Fig 4B) TNFα and/or with 20μM BAY11-7082 (BAY) respectively to activate or repress the NF-κB pathway. Results demonstrated that differences in relative transactivation potential measured in yeast were confirmed in human cells. In fact the M2 κB-RE was the most responsive, followed by RelBCons, with M1 being the least responsive. Time- and concentration-dependent TNFα responsiveness and repression by BAY were apparent. TNFα treatment led to a strong increase in p65 protein in nuclear extracts, while the p50 protein was already nuclear in mock condition and its abundance did not change significantly after treatment (Fig 4C). This observation suggests that the increase in M2 κB-RE responsiveness observed already in mock condition might be dependent primarily on p50 homodimers, while the enhanced responsiveness after TNFα treatment would be related to the increase of p65 in the nucleus.

### IκBα and the small molecules BAY 11–7082 and ethyl pyruvate inhibit NF-κB-dependent transactivation in yeast

Having established that p65 and p50TAD can act as sequence-specific TFs alone or in combination, we asked whether the assay system could be used to monitor the impact of protein or small-molecule inhibitors. In mammalian cells the functions of the canonical NF-κB pathway are mainly regulated at the level of p65 subcellular localization [42]. In particular, IκBα can sequester p65 in the cytoplasm by masking the nuclear localization sequences thereby inhibiting its translocation into the nucleus [6,7].

**Fig 5:**
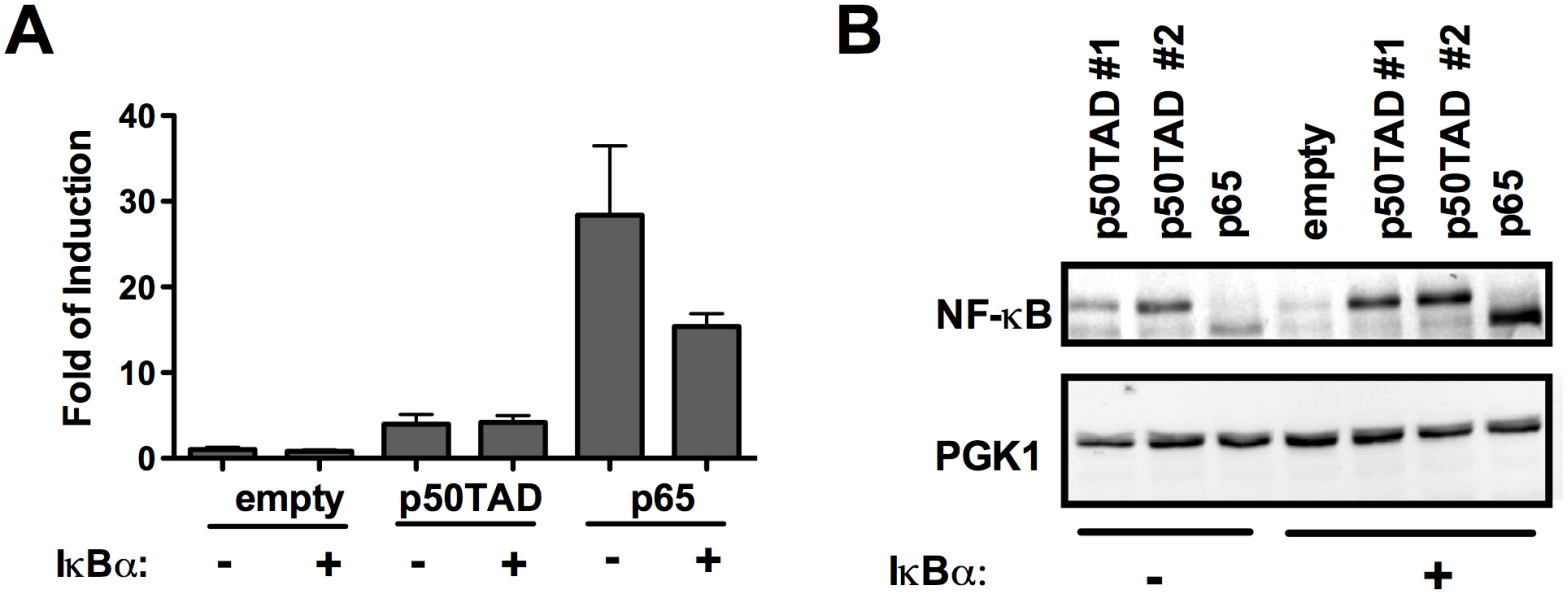
IκBα inhibits p65-dependent transactivation in yeast. The highly responsive M2 strain was used to test the impact of co-expressing IκBα with the NF-κB proteins p50TAD or p65. **A)** Luciferase assays results were obtained and plotted as described in Fig 1. Control transformants lacking the IκBα expression construct were obtained using the pRS315 empty vector. Cells were cultured in 0.032% galactose for 16 hours to achieve moderate expression of p50TAD or p65. IκBα is expressed under the constitutive *ADH1* promoter. For all conditions the light units were normalize for the optical density of the cultures. The relative luciferase activity, obtained with cells transformed with empty vectors was set to 1 and used to obtained the fold of reporter induction due to the expression of NF-κB proteins. Bars plot the average and standard errors of four biological replicates. **B)** A western blot image revealing the impact of IκBα on p50TAD or p65 protein levels. Transformants with two p50TAD expression vectors that differ for the selection marker gene (LEU2 for p50TAD #1 and TRP1 for p50TAD #2) were included. PGK1 was used as loading control.

We cloned the human IκBα cDNA into a constitutive expression vector exploiting the *ADH1* constitutive moderate promoter and the *LEU2* selection marker. This enabled us to select double transformants. The M2 κB-RE, which was the most responsive to p65 and moderately responsive to p50TAD, was chosen for these experiments. There was a significant reduction in luciferase activity with p65-IκBα double transformant cells, compared to transformants with p65 alone (Fig 5A). Interestingly, there was no effect of IκBα on the basal, constitutive level of reporter expression or on the activity of the reporter dependent on p50TAD. Immunoblots indicated that in the presence of the IκBα expression plasmid, protein levels of both p50TAD and p65 from whole cell extracts were comparable or even higher (Fig 5B), suggesting that the impact of IκBα might actually be underestimated, and that IκBα might stabilize p50TAD and especially p65 proteins by forming stable complexes as reported for mammalian cells [43,44].

**Fig 6:**
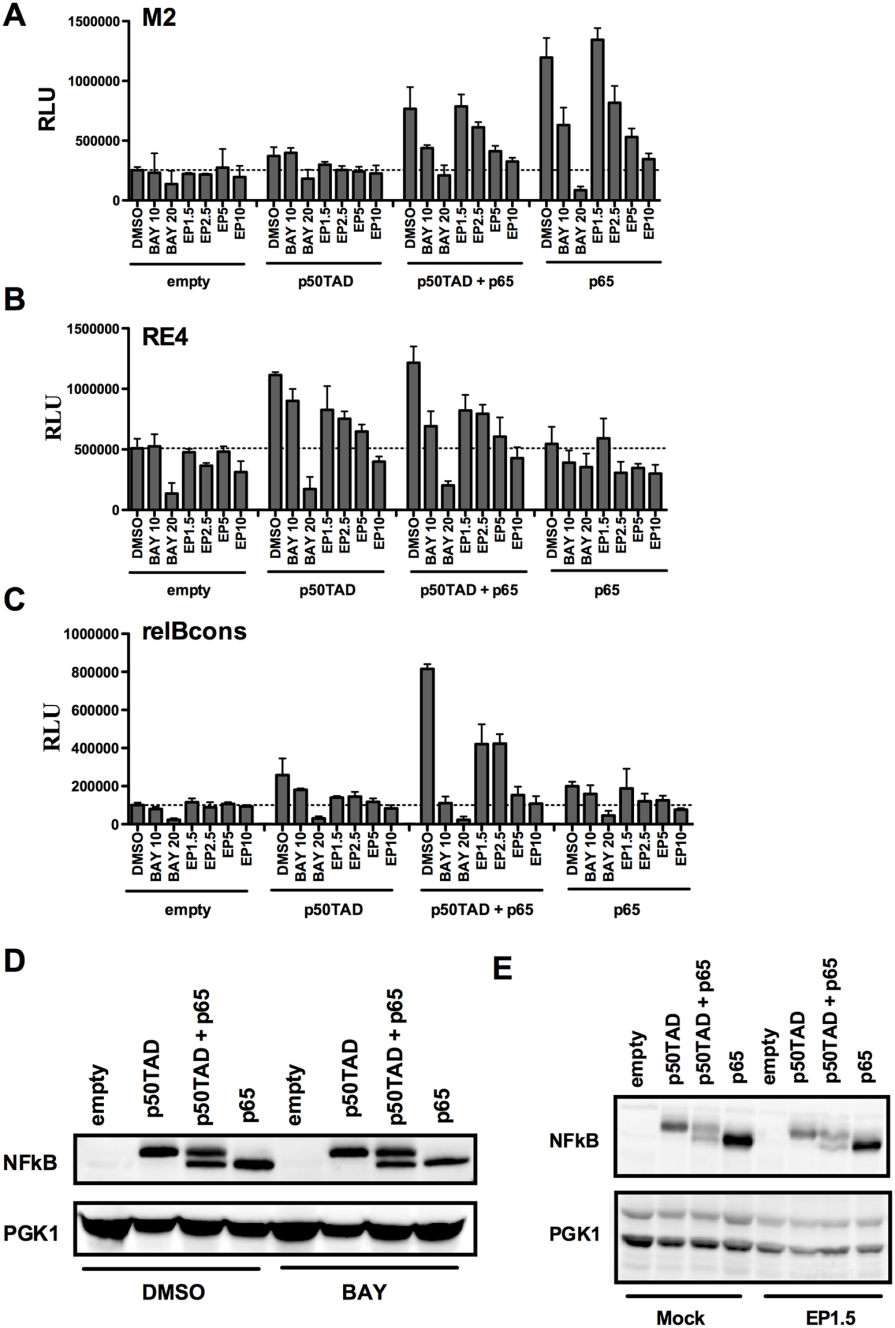
Effect of the small molecules BAY11-7082 and ethyl pyruvate on NF-κB activity in yeast. The strains containing the REs M2, RE4 and RelBCons were grown overnight (16 hours) in selective media containing low levels of galactose (0.008%) with or without the addition of different concentrations of BAY11-7082 (10μM, 20μM) and EP (1.5mM, 2.5mM, 5mM, 10mM). **A-C)** Average luciferase assays and standard errors of four biological repeats are presented. Results obtained with cells transformed with an empty expression vector are included to take into account the impact of the small molecules on the NF-κB-independent, basal expression of the reporter. **D, E)** Western blot images showing p50TAD, p50TAD + p65 and p65 protein levels in BAY and EP treated and untreated cells. PGK1 was used as a loading control.

We also explored the use of the yeast-based assay for assessing the activity of known mammalian NF-κB inhibitors BAY [9,11,45], ethyl-pyruvate (EP) [8] and parthenolide [46]. Luciferase assays were conducted in M2, RE4 and RelBCons strains as they exhibited different relative responsiveness to either p50TAD or p65 alone or to their co-expression at a low level of galactose (0.008%) (Fig 5). BAY and EP treatments led to a dose-dependent inhibition of p65-mediated transactivation. The effect was less evident with p50TAD (Fig 6). Parthenolide had no impact on p65- or p50TAD-mediated transactivation (Fig B in S1 File). As controls, the effect of the molecules on the NF-κB-independent, basal expression of the reporter, or on the steady-state levels of p65 or p50TAD proteins was examined (Fig 6D and 6E). To test the generality of the EP effect, we examined its impact on p53-mediated transcription, considering that p53 is expressed in yeast from the same *GAL1* promoter system used for NF-κB [15,30]. Unlike what was observed with p65 and p50TAD, p53 transactivation was unaffected by EP up to the 5mM dose and only slightly reduced at 10mM, while at the highest dose (20mM) it was completely inhibited. Further, no impact on p53 protein levels was seen after 2.5 or 5mM EP treatment (Fig C in S1 File). Overall, while at high doses, indirect effects could potentially bias the results, the yeast transactivation system provides a tool to study specific small molecule inhibitors of NF-κB transactivation.

## Discussion

Using a quantitative luciferase assay system, we investigated the role of κB-RE sequence in mediating NF-κB (p50 and p65) transactivation specificity. We devised a chimeric construct (p50TAD) that enabled investigations of relative transactivation specificities of p65 and p50 when expressed alone, and their functional interactions when co-expressed. We were unable to develop an equivalent assay for RelB, even though we developed a chimeric RelB-TAD construct with an equivalent approach to that used for p50 (data not shown). This lack of activity could be due to a lack of relevant cofactors in yeast or to an inability of RelB to be expressed at sufficient levels for transactivation in the yeast nucleus. Previous studies reported the development of a yeast-based assay to measure NF-κB dependent transactivation and had established that NF-κB proteins can act as sequence-specific transcription factors in yeast [38] [47]. However, our work has several unique features: variable expression of the transcription factors; creation of a chimeric, transactivation competent p50 construct; use of a chromosomally integrated reporter and a microplate format that was compatible with the analysis of small molecules potentially targeting NF-κB proteins.

p65 and p50TAD exhibited marked differences in transactivation specificity (Fig 1) and, although limited by the number of κB—REs examined, co-expression of p65 and p50TAD led to RE-selective changes in transactivation potential. Non-chimeric p50 was inactive as a transcription factor, as expected given its lack of a transactivation domain [42], but its co-expression with p65 led to strong inhibition of p65-dependent transactivation, suggesting that p50 homodimers can compete with p65 homodimers, although we cannot exclude that heterodimers containing only one transactivation domain would be inactive as a transcription factor in yeast. p65 was highly active towards 6 κB-REs, the highest being M2 (derived from the MCP-1 promoter) followed by I1 and RE4. All three contained the GGAA sequence, predicted to be a preferred binding motif (Table A in S1 File) [20,21]. Co-expression of p50TAD and p65 in yeast also demonstrated high activity with 4 κB-REs (M2, I1, RelBCons and RE4). p50TAD showed higher activity towards the RelBCons, RE5, I2 and RE4, all of which, except for I2, contained a GGGG sequence, reported to be a preferred binding motif.

**Fig 7:**
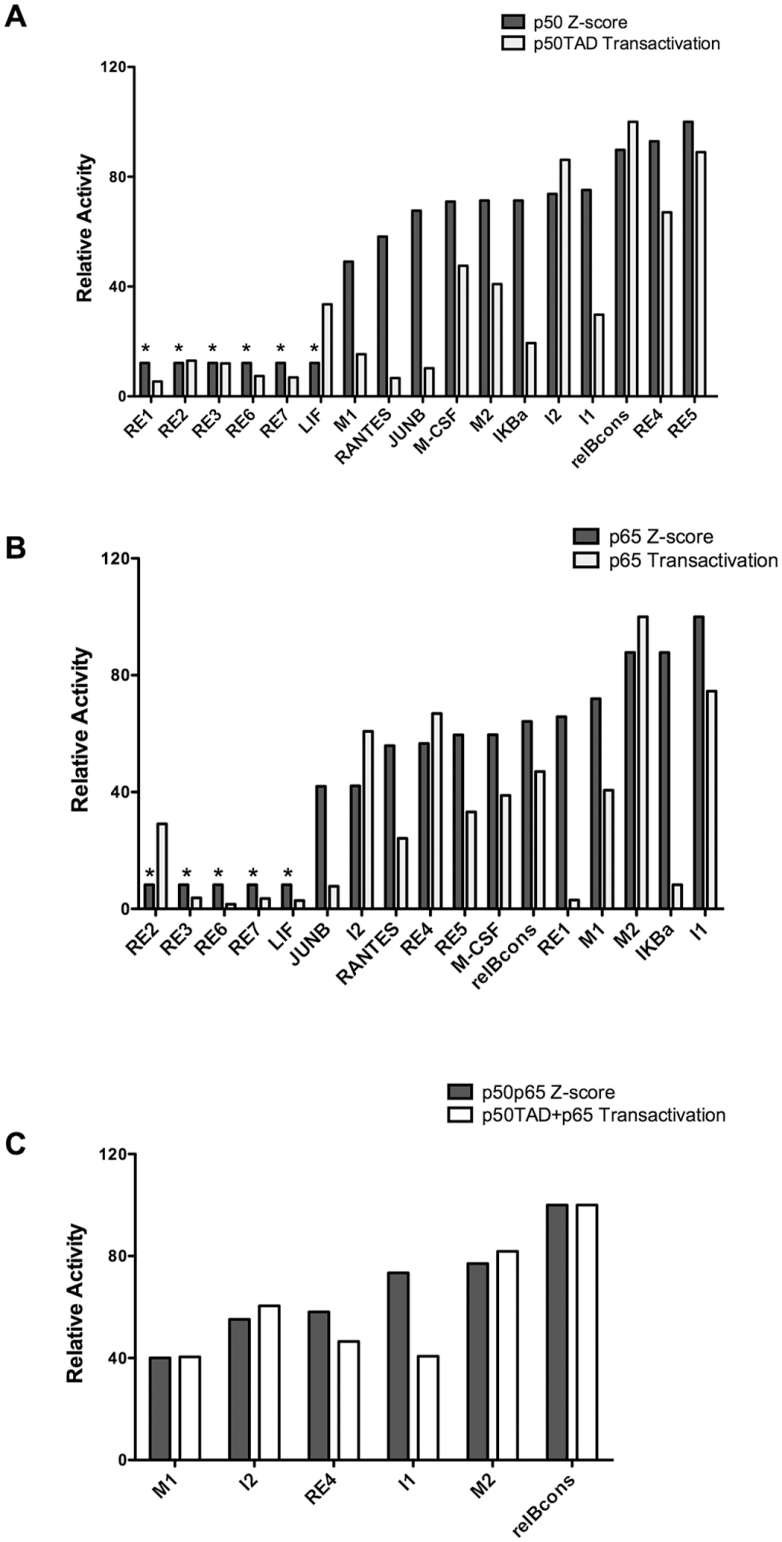
Comparison between predicted DNA binding affinity and yeast-based transactivation. **A)**, **B)** The relative binding affinities of p50 or p65 towards 17 κB-REs were obtained from (http://thebrain.bwh.harvard.edu/nfkb) and compared with the relative transactivation potential measured in yeast at moderate levels of galactose induction. REs are ordered from left to right based on increasing Z-score for DNA binding affinity. The highest affinity RE (RE5 for p50 and I1 for p65) is set to 100. REs with Z-score lower than 4, considered equivalent to background, are labeled by *. **C)** Similarly, Z-scores of the p50-p65 heterodimers and transactivation potentials are compared for the 6 κB-REs that were tested in the co-expression experiments in yeast.

We compared transactivation potentials with DNA binding affinities measured by *in vitro* gel shift assays [21] or by custom NF-κB protein binding microarray experiments and surface plasmon resonance analysis [48], as described in Fig 7. We also wanted to address various functional aspects of NF-κB target sequences, such as interactions between adjacent κB-REs and the impact of short spacers. Seventeen κB-REs were chosen for this analysis and the relative transactivation capacities were measured with p65 and p50TAD. These REs were chosen to represent a wide range of DNA binding affinities (~100-fold for p50), based on EMSA assays [21]. There were striking exceptions to an overall correlation trend between relative DNA binding affinity and relative transactivation potential both for p50 and p65+p50. For example, JunB and RE6 were extremely weak in transactivation assays but were reported to be bound with high affinity *in vitro* (Fig D in S1 File). The overall correlation was higher with DNA binding predictions based on protein-binding microarray experiments [48] that were obtained from an online tool (http://thebrain.bwh.harvard.edu/nfkb), particularly for p50/p65 heterodimers. There were however differences between the two parameters (*e*.*g*. LIF and RANTES for p50, RE2 and RE1 for p65) (Fig 7). These findings further validate our yeast-based approach. A recent study based on a largely unbiased ChIP-sequencing approach in mammalian cells confirmed that the central sequence motif of a κB-RE plays a critical role in deciding the transcriptional specificity by NF-κB proteins [22], emphasizing the significance of RE sequence even in an environment where additional *cis*-elements and trans-factors interplay to regulate transcription. Our data confirmed the impact that single nucleotide changes can have on transactivation potential (*e*.*g*. RE1 vs RE2, M1 vs M2) (Table A and Fig A in S1 File), consistent with previous reports in mammalian cells [20,21]. For example, a lentiviral-based approach demonstrated that differences in the nucleotide sequence of κB-REs embedded in about 5 kb of the MCP-1 promoter sequence could dictate the level of responsiveness to NF-κB activation by TNFα or LPS and the relative activity of different NF-κB family members [20]. In agreement with our observations in yeast, this study also established p65 as a major factor in MCP-1 promoter transactivation with a limited contribution from p50 and p52. Also, consistent with our results, the replacement of the κB-REs within MCP-1 with those present in the IP-10 promoter (I1 and I2) led to preferential responsiveness to p50 homo or hetero-dimers. However, the I1 κB-RE when tested separately exhibited a lower relative responsiveness to p50TAD in yeast.

To address the predictive value of the yeast-based assay for NF-κB-dependent transactivation in human cells expressing endogenous p65 and p50, four κB-REs were tested in gene reporter assays in MCF7 cells treated with TNFα. There was generally a good correspondence between transactivation potential in the two systems (Figs 1, 3 and 4).

Clearly, κB-RE sequences can have a direct role in influencing transactivation specificity of NF-κB proteins in a manner that is not solely related to DNA binding affinity [49]. A lack of complete correlation between DNA binding affinity and transactivation potential was also observed for the p53 family of TFs in our previous studies [50,51]. For p53, cooperative interactions between adjacent REs and a role for RE structure and kinetic properties were recognized to contribute to transactivation, especially at low expression levels [37,52]. Even a single nucleotide spacer between two half-sites that constitute the canonical p53 RE has a negative impact on p53- and, particularly, p73-dependent transactivation [51,53,54], while a spacer between two full sites has less of an effect and a short spacer could be beneficial, potentially due to steric hindrance [51].

Based on those results we studied interactions between adjacent or closely-spaced κB-REs and found somewhat more than additive, but not strong cooperative interactions between two adjacent identical κB binding half-sites. These results are consistent with a comprehensive recent study in mammalian cells that concluded that NF-κB acts non-cooperatively at closely-spaced binding sites to provide gradual increase in gene expression [41].

We used the yeast-based assay to explore the possibility to monitor the impact of protein and small molecules inhibitors on transactivation potential and specificity of NF-κB proteins. Co-expression of IκBα, a well-known inhibitor of NF-κB, led to reduced transactivation by p65. This suggests that the assay can be exploited to study crosstalk between NF-κB and upstream regulators, consistent with a previous report [38]. Interestingly, the effect of IκBα towards p50TAD was much less evident, suggesting distinct thresholds for IκBα-dependent regulation of NF-κB members. Consistently, protein-protein interaction experiments on IκBα with NF-κB p50/p65 heterodimers revealed critical interactions between IκBα and p65 [43]. We also establish that the small molecule BAY-11-7082 causes a dose-dependent inhibition of p65 as well as p50TAD-dependent transactivation. BAY11-7082 is an inhibitor of the IKK kinase that in higher eukaryotes modulates the IκBα kinase IKK. However, this molecule was shown to inhibit a broad range of protein kinases including tyrosine phosphatases [11,45]. Three different κB-REs were examined for the effect of BAY treatment. p65 homodimers or p65 co-expressed with p50TAD appeared to be more sensitive to the presence of BAY, but p50TAD homodimers were also inhibited, particularly when the RelBCons RE reporter was used. At the 20μM dose, the molecule inhibited the NF-κB-independent basal transcription of the reporter, suggesting a general repressive effect on constitutive transcription possibly due to some toxicity to yeast cells. While the mechanism of the effect of BAY-11-7082 in yeast resulting in the apparent modulation of p65 and p50 function remains to be elucidated, the effect was independent from IκBα or the IκBα kinase IKK. Notably, BAY treatment did not lead to a reduction in p65 or p50 steady state protein levels.

We also tested EP, which may directly target p65 and inhibit *in vitro* DNA binding [8]. Although it reduced both p65- and p50TAD-dependent transactivation in a dose-dependent manner, western blot analysis indicated that this effect could be due to a reduction in protein levels. However, even at high dose EP did not impact basal expression of the reporter or the growth of yeast. EP did not affect p53 transactivation up to the 5mM dose nor it impacted on p53 protein levels at 2.5 or 5mM (Fig C in S1 File). Thus, there may be a specific impact of EP on NF-κB proteins stability, but the mechanism of action of EP in our assay system remains to be established. Although parthenolide had been shown to inhibit directly and indirectly IKK [46] [55], and possibly also to directly inhibit NF-κB subunits [56], we did not observe any effect of this molecule on NF-κB-dependent transactivation in yeast.

In conclusion, our studies highlight the significance of κB-RE sequence in the transactivation specificity of NF-κB transcription factors. In fact, the transactivation abilities of p65 and p50 NF-κB proteins acting as homodimers can be distinguished with respect to κB-RE sequence specificity, and co-expression of both transcription factors can be particularly effective on selected RE sequences. Importantly, these findings with the yeast system along with the use of the p50TAD provide new opportunities to dissect the transactivation specificity of individual NF-κB protein members and the role of κB-REs, including the interaction between closely spaced REs. In addition, our results suggest that small molecules targeting NF-κB proteins can have a differential impact depending on the κB-RE being tested. Ideally this could open up the possibility to identify modifiers that can target selected NF-κB functions, for example by including in the yeast assay cofactors such as Bcl-3, CREB, p300, Tip60 that play important roles in shaping the NF-κB-directed gene regulatory network [57].

## Supporting Information

S1 File Supporting Figures and Tables. Sequence of the κB-REs tested in this study **(Table A)**. Transactivation potential of p50TAD and p65 expressed alone or together towards the M1, M2, RE4 and RelBCons κB-REs at different time points **(Figure A)**. Parthenolide has no effect on NF-κB activity in yeast **(Figure B)**. Effect of varying the concentrations of BAY11-7082 and ethyl pyruvate on NF-κB activity **(Figure C)**. A comparison between *in vitro* DNA binding affinity and relative transactivation potential of κB-REs **(Figure D)**.
(PDF)
