# Supplementary material for: Quantitative Analysis of NF-κB Transactivation Specificity Using a Yeast-Based Functional Assay
Source: PLoS One. 2015 Jul 6;10(7):e0130170. doi: 10.1371/journal.pone.0130170 (PMC4493129; doi:10.1371/journal.pone.0130170)
Supplement: S1 File — Sequence of the κB-REs tested in this study (Table A). Transactivation potential of p50TAD and p65 expressed alone or together towards the M1, M2, RE4 and RelBCons κB-REs at different time points (Figure A). Parthenolide has no effect on NF-κB activity in yeast (Figure B). Effect of varying the concentrations of BAY11-7082 and ethyl pyruvate on NF-κB activity (Figure C). A comparison between in vitro DNA binding affinity and relative transactivation potential of κB-REs (Figure D). (PDF) [file pone.0130170.s001.pdf]

Quantitative Analysis of NF-κB Transactivation specificity using a Yeast-Based Functional Assay

Vasundhara Sharma *et al.,* Supplementary Figure Legends, Figures and Table.

Fig. A. Transactivation potential of p50TAD and p65 expressed alone or together towards the M1, M2, RE4 and RelBCons κB-REs at different time points. Panels A), B) Transactivation induced by p50TAD, p65 and p50TAD+p65. Cells were grown in lower (0.008%, panel A) or higher (0.064%, panel B) levels of galactose for 3, 6 and 9 hours respectively. For each isogenic reporter strain, the luciferase activity was calculated as fold of induction with respect to the values obtained with empty vector transformants cultured and assayed in the same conditions. Presented are the average values and the standard deviations of four biological replicates.

Fig. B. Parthenolide has no effect on NF-κB-dependent transactivation in yeast. Cells were treated with two doses of parthenolide (10µM and 20µM) for 16 hours while growing in media containing two different galactose concentrations. Presented are the average values and the standard deviations of four biological replicates.

Fig. C. Effect of varying concentrations of BAY11-7082 and ethyl pyruvate on NF-κB -dependent transactivation. Panel A) M2-RE reporter cells expressing p65 cultured in high galactose (0.032%) are not inhibited by 10µM BAY11-7082, contrary to what observed when cells from the same strain were treated in lower galactose. This difference can be related to the higher levels of p65 protein. Higher dose of BAY (10µM) reduced p65- but not p50TAD-induced transactivation. High doses of ethyl pyruvate (10mM and 20 mM) completely abolished p65 and p50TAD transactivation activities without affecting the levels of NFκB-independent luciferase reporter expression (empty). **Panel B)** BAY treatment, or the lower dose of EP did not impact on p53-dependent transactivation. The human p53 cDNA is expressed from the *GAL1* promoter from a plasmid vector equivalent to the one used to express p65 or p50TAD. Panel **C)** A Western blot performed with total protein extracts from yeast cells expressing p50TAD, p50TAD+p65, p65 or p53 and treated with 2.5mM and 5mM EP showed dose-dependent reduction in NF-κB proteins (left panel), but not in p53 protein levels (right panel).

Fig. D. A comparison between *in vitro* DNA binding affinity and relative transactivation potential of κB-REs. Panel A) The relative binding affinities of p50 towards 16 κB-REs reported in a previous study [21] were compared with the relative *in vivo* transactivation potentials measured in our study. κB-REs are ordered from left to right based on predicted DNA binding affinity. The highest affinity was predicted for RE5 and set to 100 in the relative scale, while the most responsive RE in transactivation was RelBCons (also set to 100). **Panel B)** Similarly, DNA binding affinity of the p50/p65

heterodimers was compared to the transactivation data obtained in yeast with the co-expression of p50TAD and p65.

Table A: Sequence of the **κ**B-REs tested in this study.

**RE NAME**	**Sequence**
RE1	GGAAATTTCC
RE2	GGAACTTTCC
RE3	GGAAGgCTCC
RE4	GGGGAATCCC
RE5	GGGGATTCCC
RE6	GGGATACCCC
M1	GGGAACTTCC
M2	GGGAATTTCC
I1	GGGAAATTCC
I2	GGGACTTCCC
RANTES	GGGAGTTTCC
M-CSF	GGGACTTTCC
relBCons	GGGGATTTCC
IκBα	GGAAATTCCC
JUNB	GGGGCTTTCC
RE7	GGAGGATTCC
LIF	GGGGATCCCG
RE8	GGAAATTTCCGGGATACCCC
M1+M2 spacer	GGGAACTTCC-spacer-GGGAATTTCC
I1+I2 spacer	GGGAAATTCC-spacer-GGGACTTCCC
spacer sequence:	aaagctgcctcctcagagt

Fig. A

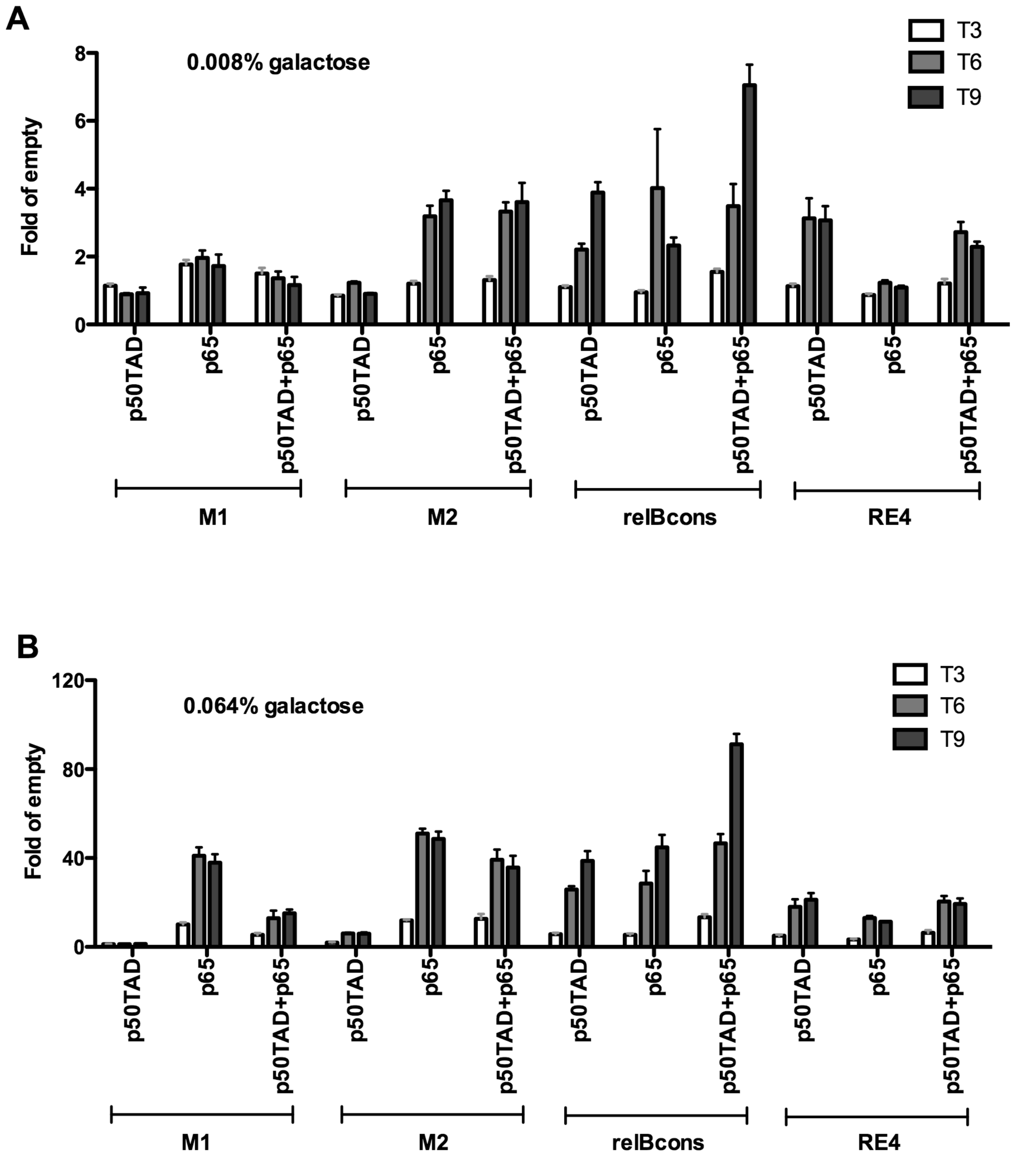

A
0.008% galactose
T3
T6
T9
Fold of empty
0
2
4
6
8
p50TAD
p65
p50TAD+p65
M1
M2
relBcons
RE4
B
0.064% galactose
T3
T6
T9
Fold of empty
0
40
80
120
p50TAD
p65
p50TAD+p65
M1
M2
relBcons
RE4

Fig. B

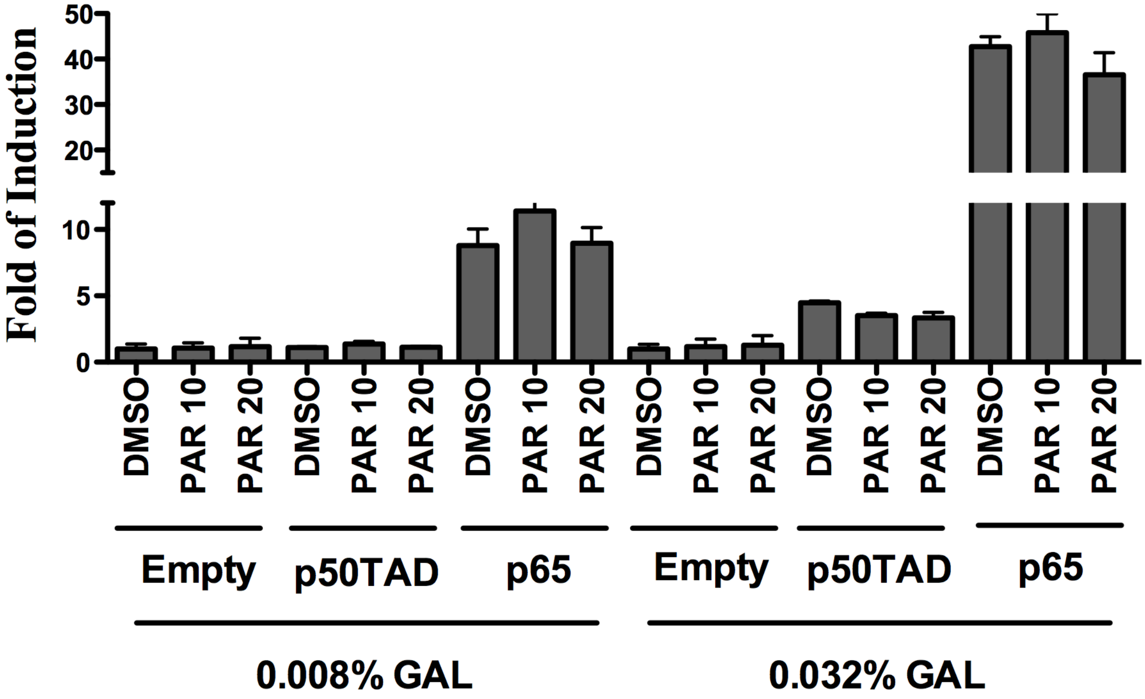

Fold of Induction
50
40
30
20
10
5
0
DMSO
PAR 10
PAR 20
DMSO
PAR 10
PAR 20
DMSO
PAR 10
PAR 20
DMSO
PAR 10
PAR 20
DMSO
PAR 10
PAR 20
DMSO
PAR 10
PAR 20
Empty
p50TAD
p65
Empty
p50TAD
p65
0.008% GAL
0.032% GAL

Fig. C

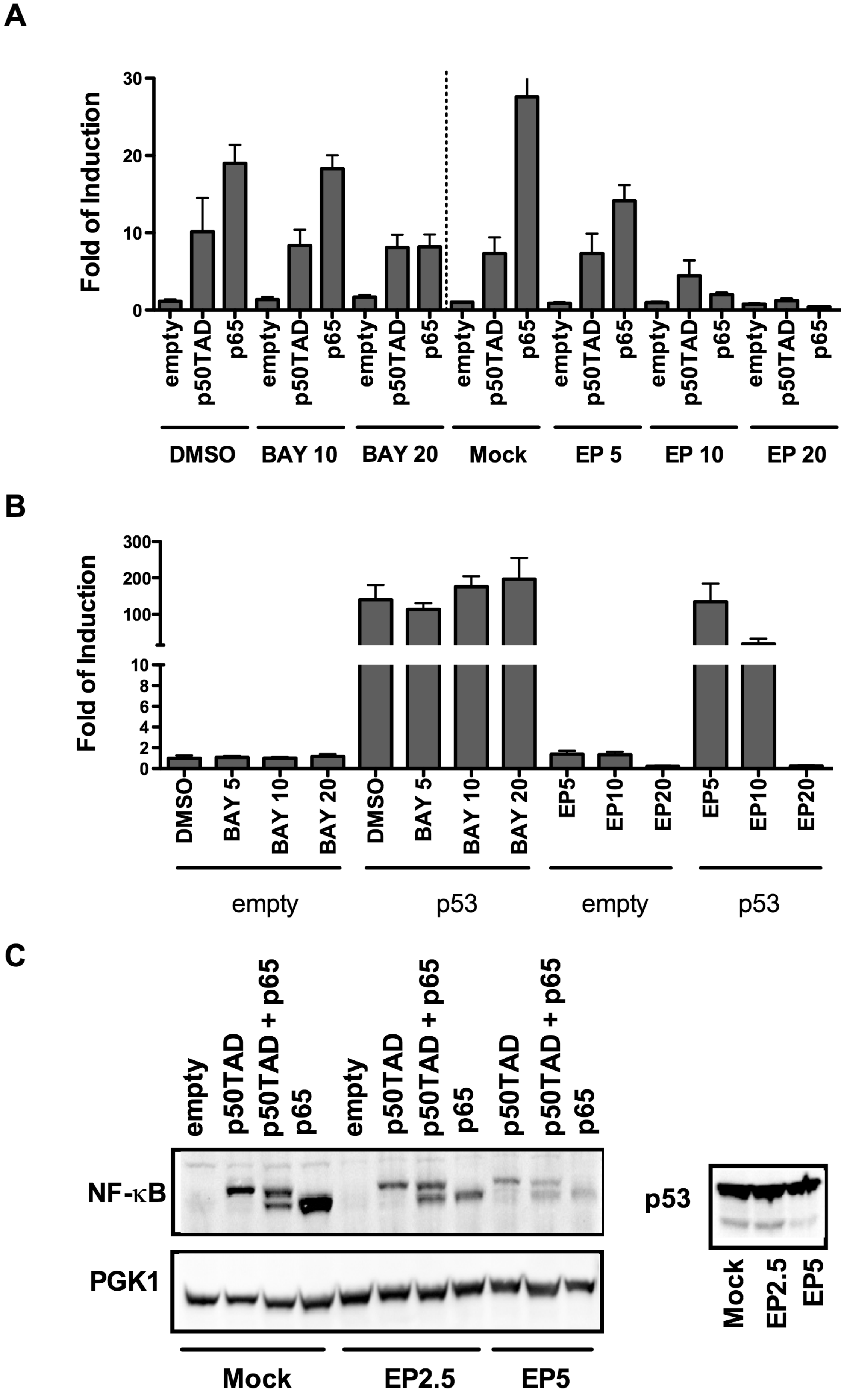
A
Fold of Induction
30
20
10
0
empty
p50TAD
p65
DMSO
BAY 10
BAY 20
Mock
EP 5
EP 10
EP 20
B
Fold of Induction
300
200
100
10
8
6
4
2
0
DMSO
BAY 5
BAY 10
BAY 20
EP5
EP10
EP20
empty
p53
empty
p53
C
empty
p50TAD
p50TAD + p65
p65
NF-κB
PGK1
Mock
EP2.5
EP5
p53
Mock
EP2.5
EP5

Fig. D

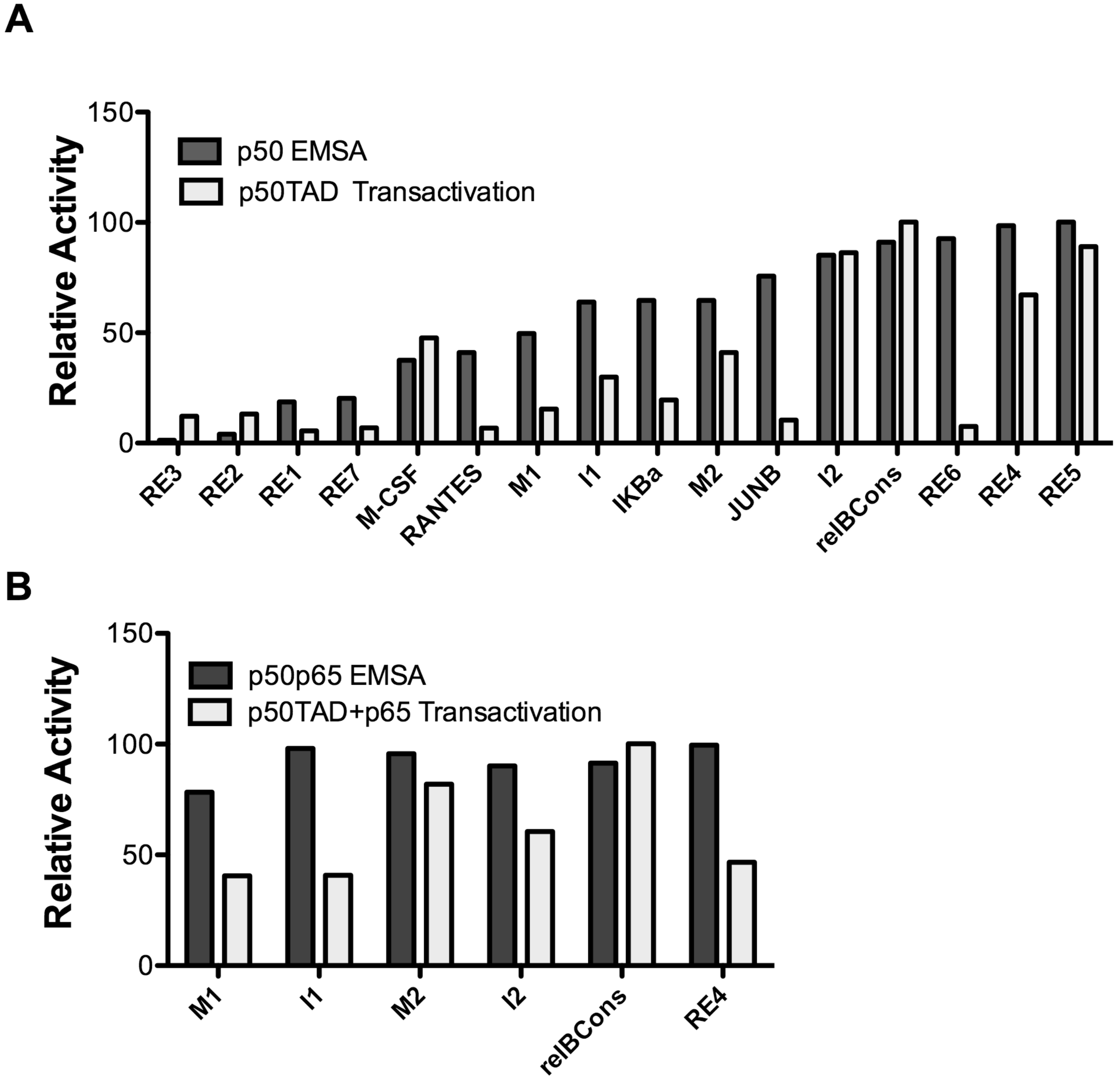
A
Relative Activity
150
100
50
0
p50 EMSA
p50TAD Transactivation
RE3
RE2
RE1
RE7
M-CSF
RANTES
M1
I1
IKBa
M2
JUNB
I2
relBCons
RE6
RE4
RE5
B
Relative Activity
150
100
50
0
p50p65 EMSA
p50TAD+p65 Transactivation
M1
I1
M2
I2
relBCons
RE4
